# Opiliones of Canada

**DOI:** 10.3897/zookeys.819.24327

**Published:** 2019-01-24

**Authors:** Jeffrey W. Shultz

**Affiliations:** 1 Department of Entomology, University of Maryland, College Park, Maryland, USA University of Maryland College Park United States of America

**Keywords:** Arachnida, biodiversity assessment, Biota of Canada, harvestmen, Opiliones

## Abstract

The taxonomic diversity of the Opiliones fauna of Canada is reviewed and summarised. At present, 36 native and seven non-native species have been documented in Canada using traditional morphological taxonomy, although more than 20 species may remain undiscovered based on species diversity in the adjacent United States and evidence from DNA barcoding. Consequently, the native fauna is yet to be fully explored and the number, distribution and ecological effects of non-native species remain unclear. Until the 1960s, work on the Canadian Opiliones fauna was largely conducted by researchers based outside the country. From that time on, several Canadian workers became active. However, these taxonomists have now retired and no one has assumed their role. Thus, there is a need to invigorate taxonomic research on the harvestmen of Canada and for the production of easy-to-use identification tools for use by non-taxonomists.

The Opiliones, or harvestmen, encompass over 6600 described species and about 50 families worldwide (Kury 2013), with 42 species representing eight families currently known from Canada (Table 1). Harvestmen are among the most common and visible groups of poorly-studied arthropod, although interest in their biology was recently invigorated by the book *Harvestmen: The Biology of Opiliones* (Pinto-da-Rocha et al. 2007). Harvestman systematics has made significant progress in the last 20 years, after more than a century of neglect. As with many invertebrate groups, the rate of taxonomic progress suffers from a shortage of active taxonomists. This is particularly true in Canada, where the last major Canadian harvestman worker, Robert Holmberg, has retired.

**Table 1.** Census in Opiliones in Canada.

Taxon^1^	No. species reported in Dondale (1979)^2^	No. species currently known from Canada^3^	No. BINs^4^ available for Canadian species	Est. no. undescribed or unrecorded species in Canada	General distribution by ecozone^5^	Information sources
**Suborder Cyphophthalmi**						
Sironidae		0	0	2	Pacific Maritime, Montane Cordillera	Bragg and Holmberg 2009, Giribet and Shear 2010
**Suborder Laniatores**						
Paranonychidae		2	1	2	Pacific Maritime, Montane Cordillera	Briggs 1971, Shear and Derkarabetian 2008, Bragg and Holmberg 2009, Derkarabetian and Hedin 2014
Travuniidae		0	0	1	Mixedwood Plains	Bishop 1949
**Suborder Dyspnoi**						
Ischyropsalididae		0	0	1	Pacific Maritime	Bragg and Holmberg 2009, Richart and Hedin 2013
Sabaconidae		3	6	3	Pacific Maritime, Boreal Plain, Boreal Shield?, Mixedwood Plains; Atlantic Maritime	Cokendolpher and Lee 1993, Holmberg 1999, Holmberg and Buckle 2004
Taracidae		5	2	1	Pacific Maritime, Montane Cordillera, Mixedwood Plains	Bragg and Leech 1972, Cokendolpher and Lee 1993, Brousseau 2011, Shear and Warfel 2016
Nemastomatidae		3 (1)	4	1	Pacific Maritime, Mixedwood Plains, Atlantic Maritime	Bragg and Holmberg 2009, Shear 2016
Acropsopilionidae		1	0	0	Mixedwood Plains, Boreal Shield, Atlantic Maritime?	Cokendolpher and Lee 1993, Shultz 2013
**Suborder Eupnoi**						
Caddidae		1	2	1	Mixedwood Plains, Boreal Shield, Atlantic Maritime	Cokendolpher and Lee 1993, Shultz 2013
Phalangiidae		12 (6)	16	4	all ecozones	Bragg and Holmberg 1974, 2009, Cokendolpher 1981b, [Bibr B12][Bibr B14], Shear 2016, Cokendolpher and Holmberg in press
Protolophidae		1	2	1	Pacific Maritime	Bragg and Holmberg 2009
Sclerosomatidae		15	31	5	Pacific Maritime, Montane Cordillera, Boreal Plains, Prairies, Boreal Shield, Mixedwood Plains, Atlantic Maritime	Davis 1934, Katayama and Post 1974, Cokendolpher 1981a, Cokendolpher and Lee 1993, Ingianni et al. 2011, Hogue 2015
**Total**	**47**	**43 (7)**	**64**	**22**		

**^1^**Classification follows Kury (2013), with subsequent modifications by Schönhofer (2013) and Groh and Giribet (2015). **^2^**Dondale (1979) did not report the number of species at lower taxonomic levels. ^3^Numbers in parentheses represent the number of non-native species included in the total. **^4^**Barcode Index Number, as defined in Ratnasingham and Hebert (2013). ^5^See figure 1 in Langor (2019) for a map of ecozones.

In Danks’ seminal survey of Canadian terrestrial arthropods (Danks 1979), Dondale (1979) summarised the status of harvestman systematics in Canada in fewer than 60 words and without literature citations. He suggested that there were approximately 50 species in the country, which may be close to the true total (Table 1). At the time, he judged that taxonomic instability within the order was too great to assign species reliably to families or to other higher taxa. Since then, harvestman taxonomy has improved greatly, although insights from molecular phylogenetic studies continue to inspire reorganisations at the subfamilial, familial, and even subordinal levels (e.g., Hedin et al. 2012, Schönhofer 2013, Groh and Giribet 2015).

The rate of discovering and cataloguing Canadian harvestmen has been a slow and largely international process that has tended to lag behind that of other western countries. The year in which each native species was first recorded, data mined primarily from Cokendolpher and Lee (1993), illustrates several trends. For each of 24 species native to both Canada and the USA, the first record from the USA preceded the first record from Canada by about 60 years; that is, 53.6 ± 37.4 (SD) years (range: 9-149 y). Only two species were discovered first in Canada, with a difference of 34.5 ± 2.1 years. For over a century (1860 to 1966), all first national records in Canada were established by non-Canadian workers, most from the USA (Banks 1902, 1916, Crosby 1907, Davis 1934, Crosby and Zorsch 1935, Bishop 1949), with the remainder based in Europe (Britain: Walker 1860; Germany: Roewer 1910, 1957; Switzerland: Schenkel 1951; Finland: Hackman 1956). Canadian workers mobilised in earnest in the 1960s and 1970s, with notable contributions from Judd (1966–1978) in Ontario and Bragg and Leech (1972) in British Columbia. Harvestman systematics in western Canada continued to benefit from the work of Phillip Bragg, Robert Holmberg and collaborators (Bragg and Holmberg 1974, 2009, Holmberg 1999, Holmberg et al. 1981, Holmberg and Buckle 2004, Holmberg and Cokendolpher 1997). In contrast, the harvestman fauna of the central and eastern provinces has been comparatively neglected. In fact, significant first national records in the east have been made by citizen scientists contributing photos to such web sites as BugGuide (https://bugguide.net), including first national records for *Crosbycusdasycnemus* (Crosby) by Brousseau (2011) and *Leiobunumnigropalpi* (Wood) by Hogue (2015).

The Opiliones are divided into four suborders with the following relationships: Cyphophthalmi, (Laniatores, (Dyspnoi and Eupnoi)). Thus, the current taxonomic hierarchy does not strictly reflect the generally accepted phylogeny. Laniatores, Dyspnoi, and Eupnoi form the clade Phalangida, which is the sister group to Cyphophthalmi, and the Dyspnoi and Eupnoi are united in the Palpatores, which is the sister group to Laniatores (Kury 2018).

The Cyphophthalmi, or mite harvestmen, are not known from Canada, although Bragg and Holmberg (2009) suggested that *Sirokamiakensis* (Newell) and *S.acaroides* (Ewing), with northern Washington populations of the latter now known as *S.boyerae* Giribet & Shear, 2010, might range into southern British Columbia. Global biogeographic patterns indicate that Sironidae is the only family likely to occur in Canada (Boyer et al. 2007).

The taxonomic diversity of the Laniatores, or armored harvestmen, is very high in the New World tropics and subtropics but diminishes significantly with increasing latitude (Kury 2003). The recently circumscribed Paranonychidae encompass the former triaenonychid subfamilies Paranonychinae and Sclerobuninae and is the only family likely to occur in western Canada. With the synonymisation of *Sclerobunusparvus* Roewer with *Paranonychusbrunneus* (Banks) (Shear and Derkarabetian 2008), just two species are known from British Columbia. Two others known from the northern USA might extend into that province as well (Bragg and Holmberg 2009). Bishop (1949) recorded *Erebomasterflavescens* (Cope) (Cladonychiinae: Travuniidae) in southeastern New York, an observation that is often overlooked (e.g., Kury 2003). Given the great distance from its known congeners in the mid-Atlantic and mid-western USA, *Erebomaster* may be more widespread than is currently supposed, and might even range into southeastern Canada.

The morphologically diverse Holarctic suborder Dyspnoi contains three main lineages, Ischyropsalidoidea, Troguloidea, and Acropsopilionidae, with the latter recently transferred from Caddoidea (Eupnoi) (Groh and Giribet 2015). The family-level classification of Ischyropsalidoidea has undergone significant reorganisation (Schönhofer 2013). Sabaconidae now includes only *Sabacon* (not *Taracus*), with *Sabacon* species being widespread in Canada and with barcoding data suggesting the existence of greater species diversity than current taxonomy would suggest (Table 1). The Ceratolasmatidae, erected by Shear (1986), was disbanded and its components transferred to the new family Taracidae (*Crosbycus*, *Hesperonemastoma*, *Oskoron*, *Taracus*) and to the subfamily Ceratolasmatinae (*Acuclavella*, *Ceratolasma*) within the family Ischyropsalididae, which otherwise contains only the European *Ischyropsalis*. Among the four families in Troguloidea, only the Nemastomatidae occur in Canada, specifically the native ortholasmatines, *Dendrolasma* and *Ortholasma*, in British Columbia (Bragg and Holmberg 2009), and the non-native European nemastomatine, *Nemastomabimaculatum* (Fabricius), in the East (Shear 2016). Only one acropsopilionid species, *Acropsopilioboopis* (Crosby), is known from Canada and no additional species are expected to occur there.

The suborder Eupnoi consists of two superfamilies, the species-poor Caddoidea, and the species-rich Phalangioidea. Caddoidea s. str. (Groh and Giribet 2015) contains one genus, *Caddo*, with two species. *Caddoagilis* Banks is known from southeastern Canada and *C.pepperella* Shear may eventually be found there, given its occurrence in New England and its recent discovery in northern Wisconsin (Shultz 2013). The family-level taxonomy within Phalangioidea is in flux, with three major lineages being relevant to the Canadian fauna: Phalangiidae (25% of known Canadian species), Sclerosomatidae (36% of known Canadian species), and at least one species of Protolophidae (Bragg and Hoffman 2009, Ratnasingham and Hebert 2013: Barcode Index Numbers (BINs) BOLD:ACJ0890, BOLD:ACJ0891). Six phalangiid species are native to Canada: *Mitopusmorio* (Fabricius), *Odielluspictus* (Wood), *Leptobunusborealis* Banks, *Leptobunusparvulus* (Banks), *Liopilioyukon* Cokendolpher, and *Liopilioglaber* Schenkel, and at least six appear to have been introduced from Europe: *Oligolophustridens* (CL Koch), *Paroligolophusagrestis* (Meade), *Opilioparietinus* (DeGeer), *Phalangiumopilio* Linneaus, *Lophopiliopalpinalis* (Herbst), and *Rilaenatriangularis* (Herbst) (Shear 2016, Cokendopher and Holmberg 2018). The leiobunine Sclerosomatidae are represented by five native genera: *Hadrobunus* (2 spp.), *Leiobunum* (9 spp.), *Leuronychus* (1 sp.), *Nelima* (2 spp.), and *Togwoteeus* (1 sp.).

Increases in the number of species known from Canada are most likely to come from four sources: range extensions into Canada, introductions, taxonomic revisions, and discovery of cryptic species. The discovery of native species already known from the adjacent USA is the most likely source of new Canadian records. Bragg and Holmberg (2009) listed six species currently known in the USA that might extend into British Columbia, although the taxonomy of several species has since changed and a targeted search for northern populations of one was unsuccessful, i.e., *Acuclavella* in Richart and Hedin (2013). An unusually thorough study of harvestman distributions in North Dakota (Katayama and Post 1974) showed *Eumesosomaroeweri* (Goodnight and Goodnight) and *Trachyrhinus* [*T.favosus* (Wood) and/or *T.marmoratus* Banks] (see Cokendolpher 1981) to be present in counties along the USA-Canadian border. This would add two genera and one subfamily (Gagrellinae) to the Canadian fauna. Canada also seems prone to the introduction and establishment of European harvestmen, especially phalangiids (Cokendolpher and Holmberg 2018). The presence of the European *Trogulustricarinatus* (Linneaus) in New York and Massachusetts (Shear 2016) indicates that a similar introduction could occur in Canada. Taxonomic revisions can also increase or decrease species diversity. Shear and Warfel (2016) recorded two new Canadian *Taracus* species and a new genus, *Oskoron*, that might extend into Canada. Also, an ongoing revision of *Hadrobunus* (Sclerosomatidae) has revealed two species in Ontario (J Shultz unpubl. data), where only one was assumed to exist. On the other hand, in *Leiobunum* (Sclerosomatidae), *L.formosum* (Wood) and *L.nigripes* Weed were found to be junior synonyms of *L.verrucosum* (Wood) (Shultz 2008), which eliminated two nominal species from the Canadian fauna. Finally, genetic diversity revealed by analysis of molecular genetic data, including barcodes (e.g. Ratnasingham and Hebert 2013), may indicate the existence of cryptic species (Table 1).

Progress in the discovery and understanding of harvestman diversity in Canada will require the effort of one or more professional taxonomists to engage in active, modern research on the order. A particularly urgent goal is the production and distribution of accessible and easy-to-use tools for the identification of the harvestmen species known or likely to occur in Canada. The virtual absence of such resources has already had significant negative consequences. For example, of the 64 BINs of Canadian harvestmen in the Barcodes of Life Data systems (BOLD) (Ratnasingham and Hebert 2013), about 25% were considered either “unidentified”, although they could be readily identified from photos of voucher specimens (ten BINs), or were identified incorrectly (six BINs). Indeed, the entry for *Leiobunumvittatum* (BOLD:AAM8191) contains no specimens of that species but encompasses at least three morphologically and geographically distinct species of *Hadrobunus*. Lastly, specimens in many BINs are identified to genus or species when, in fact, the photos show either juveniles or otherwise unidentifiable specimens. In some cases, the ambiguous specimens are determined as European adventives that are otherwise unrecorded from Canada, a situation that, if correct, could suggest an early stage in the expansion of a potential invasive species. In fact, results from barcoding based on accurate identifications (BOLD:AAM8194) revealed a previously unknown introduction and expansion of the European *Oligolophustridens* in Alberta, Saskatchewan and extreme southeastern British Columbia. Clearly, surveys of the harvestman fauna should be undertaken throughout the country to establish the species composition of the native fauna as well as the distribution and environmental impacts of the comparatively numerous species that have been introduced into Canada.
